# Caloric restriction reveals a metabolomic and lipidomic signature in liver of male mice

**DOI:** 10.1111/acel.12241

**Published:** 2014-07-23

**Authors:** Mariona Jové, Alba Naudí, Omar Ramírez-Núñez, Manuel Portero-Otín, Colin Selman, Dominic J Withers, Reinald Pamplona

**Affiliations:** 1 Department of Experimental Medicine, University of Lleida-Biomedical Research Institute of LleidaLleida, 25198, Spain; 2 Institute of Biodiversity, Animal Health and Comparative Medicine, University of GlasgowGraham Kerr Building, Glasgow, G12 8QQ, UK; 3 Metabolic Signaling Group, Medical Research Council Clinical Sciences Centre, Imperial College LondonLondon, W12 0NN, UK

**Keywords:** caloric restriction, glycolytic pathway, phospholipids oxidation, protein oxidative damage, tryptophan pathway

## Abstract

Lipid composition, particularly membrane unsaturation, has been proposed as being a lifespan determinant, but it is currently unknown whether caloric restriction (CR), an accepted life-extending intervention, affects cellular lipid profiles. In this study, we employ a liquid chromatography quadrupole time-of-flight-based methodology to demonstrate that CR in the liver of male C57BL/6 mice: (i) induces marked changes in the cellular lipidome, (ii) specifically reduces levels of a phospholipid peroxidation product, 1-palmitoyl-2-glutaryl-sn-glycero-3-phosphatidylcholine, (iii) alters cellular phosphoethanolamine and triglyceride distributional profiles, (iv) affects mitochondrial electron transport chain complexes, increasing complex II and decreasing complex III and (v) is associated with specific changes in liver metabolic pathways. These data demonstrate that CR induces a specific lipidome and metabolome reprogramming event in mouse liver which is associated with lower protein oxidative damage, as assessed by mass spectrometry-based measurements. Such changes may be critical to the increased lifespan and healthspan observed in C57BL/6 mice following CR.

## Introduction

Membrane fatty acid composition appears to play an important role in the aging process and in the determination of interspecies longevity (Pamplona *et al*., 2002; Hulbert *et al*., 2007; Pamplona, 2008). Likewise, mitochondrial lipids, including phosphatidylcholines (PC) and phosphatidylethanolamines (PE), show a similar relationship, with longer life being correlated with a lower incidence of fatty acid double bonds (Mitchell *et al*., 2007). Fatty acids differ dramatically in their susceptibility to peroxidation. Saturated fatty acids (SFAs) and monounsaturated fatty acids (MUFAs) are highly resistant to peroxidation by physiological oxidants. In contrast, polyunsaturated fatty acids (PUFAs) are particularly susceptible, with the higher the unsaturation index of a fatty acid molecule the higher its susceptibility to peroxidation (Hulbert *et al*., 2007). Several studies also report that the level of unsaturation in membrane phospholipids increases in an age-dependent manner (Lambert *et al*., 2004; Laganiere & Yu, 1993; see review Naudí *et al*., 2013). Furthermore, longer lived animals (both vertebrates and invertebrates) exhibit a lower degree of membrane fatty acid unsaturation and are relatively resistant to *in vivo* and *in vitro* lipid peroxidation (LPO) (Pamplona *et al*., 1996, 1998; Hulbert *et al*., 2006; see review Naudí *et al*., 2013). This LPO resistance in tissues and mitochondrial proteins from long-lived animals leads to a lower steady-state level of lipoxidation-derived adducts formed by the interaction of proteins and DNA with LPO-derived soluble intermediates, e.g. oxysterols, hydroperoxides, endoperoxides and reactive carbonyl components (Zimniak, 2011; Naudí *et al*., 2013). While these species could act as physiological mediators, the progressive accumulation of their derived modifications is associated with tissue and cellular dysfunction, and are a typical feature of aging and of various pathologies (Pamplona, 2011).

Caloric restriction (CR) has been shown to increase both median and maximum lifespan in many organisms (Minor *et al*., 2010; Selman, 2014) although the positive effects of CR on lifespan do not appear to be universal (Cooper *et al*., 2004; Swindell, 2012; Selman, 2014). CR also induces a range of beneficial health benefits in many organisms including humans (Mercken *et al*., 2013), and it would appear, in some instances, that significant health benefits can exist without lifespan extension (Selman, 2014). While the effects of CR on lifespan and healthspan have been known for nearly a century, the precise molecular mechanisms underlying these effects remain unknown, although a whole range of putative mechanisms have been proposed (Anderson & Weindruch, 2010; Xiang & He, 2011). For example, oxidative damage to biomolecules has been suggested as being a key mechanism underlying aging (Gredilla & Barja, 2005; Naudí *et al*., 2013), and that CR may act by directly reducing molecular oxidative damage. Taking into account the role of lipids in membrane characteristics and in aging (see above), CR seems to trigger an adaptive response protecting membrane integrity (Naudí *et al*., 2013). Thus, CR might delay aging and extend longevity through mechanisms that involve changes in the lipoxidative status (Gredilla & Barja, 2005; Yang & Hekimi, 2010; Kowaltowski, 2011; Ristow & Schmeisser, 2011).

Due to the relationship of lipids with aging (e.g. changes in membrane fatty acid composition and membrane order parameters, increased levels of membrane LPO and lipoxidative products accumulation) (Hulbert *et al*., 2007; Naudí *et al*., 2013), it is feasible to postulate that some of the life and health extending effects of CR would lead to age-resistant profiles in lipid composition. Given that currently little is known on how CR impacts on the lipidome, we characterized the impact of CR on the liver lipidome in male C57BL/6 mice by performing mass spectrometry-based lipid analyses. Increasing evidence suggests that different phospholipid classes induce specific biological effects and that nonrandom oxidation can occur between phospholipids. So, to understand the specific cellular species responsible for the general change in peroxidizability and saturation levels and to evaluate potential effects in other lipid classes, we focused on phospholipids and other esterified lipids. We show that CR significantly altered the hepatic lipidome and metabolome in male C57BL/6 mice. A variegated change in the relative abundance of specific triglycerides (TAG) and PE was apparent as well as alterations in energy metabolism and tryptophan oxidation pathways. We suggest that these specific changes may be the result of a metabolic reprogramming leading to lower levels of oxidative damage which could contribute to the increased lifespan of CR mice.

## Results

### CR induces marked changes in liver lipidome

**Figure 1:**
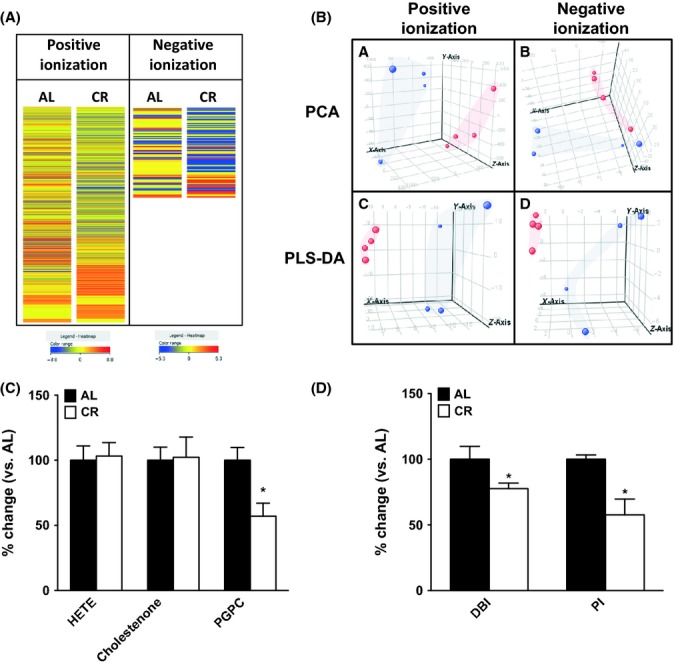
CR induces specific and marked changes in liver lipidome. (A) Heat map showing the molecular features (see main text for definition) found in liver from *ad libitum* (AL) and 30% caloric restriction (CR) mice in lipidomic analysis. Each line of this graphic represents an accurate mass ordered by retention time, coloured by its abundance intensity normalized to internal standard and baselining to median/mean across the samples. Individual scale maps for heat intensity are shown below each sample type. The scale from blue (lower abundance) to red (higher abundance) represents this normalized abundance in arbitrary units. *n* = 4 samples per group. (B) Principal component analysis (PCA) and Partial Least Square Discriminate Analysis (PLS-DA) graphs demonstrating the differentiation effect of diet in lipidomic profiles. Red spots represent samples from AL group and blue spots CR group. Explained variability of each representation in PCA is 61.26% (positive ionization) and 68.96% (negative ionization). Overall accuracy in PLS-DA analysis is 0.88 (positive ionization) and 1 (negative ionization). X: Principal component 1, Y: Principal component 2, Z: Principal component 3. *n* = 4 samples per group. (C) CR does not induce any changes in the arachidonic acid oxidation product hydroxyeicosatetraenoic acid (HETE) and the oxysterol-5-cholesten-3β-ol-7-one (cholestenone). CR decreases the phospholipid oxidation product 1-palmitoyl-2-(5-oxo valeroyl)-*sn*-glycero-3-phosphatidylcholine (PGPC) derived from 1-palmitoyl-2-arachidonoyl-sn-glycero-*3*-phosphocholine in liver. Values shown are means ± SEM from *n* = 4 samples per group. (D) CR induces changes in fatty acid composition leading to significantly lower peroxidizability index (PI) and double bond index (DBI) (AL values: for DBI: 162.50 ± 0.87; for PI: 145.50 ± 4.40). Values shown are means ± SEM from *n* = 4 samples per group. *P < 0.05 with respect to AL group by Student’s *t*-test. AL, ad libitum; CR, Caloric restriction.

We initially examined how exactly CR modified the liver lipid profile relative to age-matched *ad libitum* (AL) fed control mice, using both a nontargeted and a targeted approach. Employing a nontargeted approach (Sana *et al*., 2010) we detected 1706 lipid species (1608 in positive and 98 in negative ionization) altered in the liver lipidome (Fig. 1A) of CR mice. We then explored the hepatic metabolic profiles of AL and CR groups further by employing multivariate statistical analyses (Trygg *et al*., 2007), including principal component analysis (PCA) (unsupervised) and partial least-squares discriminate analysis (PLS-DA) (supervised) (Fig. 1B). The PCA analysis explained ~60–70% of the sample variability indicating that it was a good approximation of the effects of CR in the liver lipidome. Both the supervised and unsupervised approaches showed a good clustering of the samples according to diet (AL or CR), indicating that diet explained the majority of this variability. Statistical analysis revealed that 101 lipid species in the liver differed between AL and CR animals (Data S1). Among these, 11 altered by CR were associated with specific glycerophospholipids and glycerolipids (Table 1). However, CR had no impact on other lipid families (e.g. sphingolipids, prenol lipids), on free fatty acids (stearic acid, linoleic acid, palmitic acid, arachidonic acid, oleic acid, myristic acid, docosahexaenoic acid) or on cholesterol levels (Fig. S1). Specifically, the analysis revealed that PE (32:0, 35:0, 34:0, 40:6 and 38:2) were increased, whereas another PE (35:0) and the phosphoserine (36:0) were decreased in CR animals compared to AL mice. Concerning glycerolipids, one diacylglycerol (DAG(50:7)) and two TAG (48:0 and 62:1) were decreased and the TAG (56:2) was increased in CR animals. Total levels of TAG were measured using a colorimetric levels and a slightly decrease was observed after CR diet (Fig. S2A). On the other hand, the total cholesterol content was not affected (Figs S1 and S2B) by CR suggesting a lipid-specific effect of CR diet.

**Table 1:** Differential lipid molecules identified

Classification (Lipid maps)	Compound	Regulation	Fold change (respect AL)	*P*
Glycerophospholipids	PE(40:6)	up	1–5	0.004
	PE(38:2)	up	>10	0.009
	PE(35:0)	up	1–5	3.54 E-5*
	PE(35:0)	down	1–5	0.02
	PE(34:0)	up	1–5	0.045
	PE(32:0)	up	>10	8.4 E-9*
	PS(36:0)	down	>10	3.13 E-10*
Glycerolipids	DAG(50:7)	down	>10	0.026
	TAG(62:1)	down	>10	0.019
	TAG(56:2)	up	>10	0.04
	TAG(48:0)	down	1–5	0.036

* Correction for false discovery rate by Benjamini–Hochberg test.

AL, *ad libitum*.

### CR lowered hepatic 1-palmitoyl-2-glutaryl-sn-glycero-3-phosphatidylcholine (PGPC) content, a specific product of phospholipid peroxidation

We then analysed specific LPO markers in the liver of AL and CR mice by using targeted lipidomics. Using this approach we detected and quantified three different LPO markers: hydroxyeicosatetraenoic acid (HETE), 5-cholesten-3β-ol-7-one and PGPC. Only PGPC levels were decreased in CR mice, again suggesting a glycerophospholipid -specific effect (Fig. 1C). Lower PGPC levels, a specific phospholipid oxidation product, in CR mice led us to examine the susceptibility of hepatic cell membranes to peroxidation. Analysis of fatty acid after transesterification revealed that CR altered the fatty acid composition of total lipids in liver (Table S1). Specifically, CR significantly decreased the total number of double bonds (DBI) and the peroxidizability index (PI) (Fig. 1D) in liver through decreasing PUFA levels, increasing MUFA levels, while unsaturated fatty acids (UFAs) remained unchanged. Therefore, the changes observed in fatty acid membrane composition may help explain the lower levels of oxidative damage we observed in our CR cohort.

### CR affects autophagy and ubiquitin-dependent proteolysis

**Figure 2:**
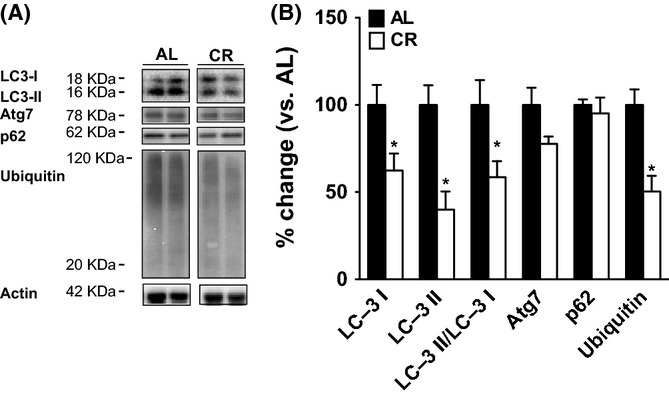
CR affects autophagy and protein degradation components in liver. (A) Representative immunoblots showing relative levels of mitrotubule-associated protein light chain 3 I (LC3-I), LC3-II, autophagy protein 7 (Atg7), p62 and ubiquitin. (B) Relative amounts of LC3-I, LC3-II, LC3-II/LC3-I, Atg7, p62 and ubiquitin immunoreactivity in liver from AL and CR mice after densitometric analyses. Values shown are means ± SEM from *n* = 4 samples per group. The isoform b of the protein LC3 was detected. *P < 0.05 with respect to AL group by Student’s *t*-test. AL, ad libitum; CR, Caloric restriction.

The lower levels of phospholipid oxidation and the increased levels of PE found in CR mice could also be explained by an increase in lipid repair mechanisms (e.g. autophagy). It is well established that specific lipid species participate in the formation of the vacuolar compartments involved in autophagy, and lipid-modifying enzymes modulate the dynamics of these compartments (Rodriguez-Navarro & Cuervo, 2010). Consequently, we analysed the macroautophagy-associated proteins autophagy protein 7 (Atg7), p62 and both microtubule-associated protein light chain 3 I (LC3-I) and LC3-II (isoform b). In addition, we examined the level of ubiquitination of hepatic proteins. In CR mice, the levels of LC3-I and LC3-II were reduced as was the LC3-II/LC3-I ratio, although neither Atg7 nor p62 were affected by treatment (Fig. 2). Liver ubiquitin levels were also decreased in CR mice suggesting greater proteolytic activity or lower protein damage. This then led us to examine the levels of specific protein oxidation markers.

### CR reduces the steady-state levels of protein oxidative damage

**Figure 3:**
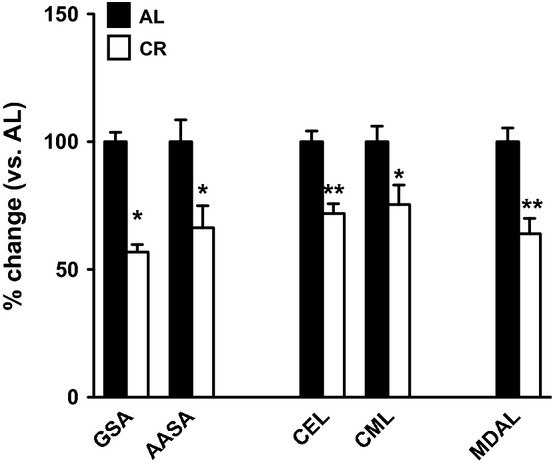
Proteins from CR samples show significant decrease in the steady-state levels of oxidative damage markers: glutamic semialdehyde (GSA), aminoadipic semialdehydes (AASA) (markers of protein direct oxidation), carboxyethyl-lysine (CEL) (arising from glycoxidation), carboxymethyl-lysine (CML) (from both lipoxidation and glycoxidation damage) and malondialdehydelysine (MDAL) (originated from lipoxidation). Values shown are % changes of mean ± SEM over values from AL group (AL group values are: GSA: 6293.36 ± 231.21 μmol/mol lysine; AASA: 193.63 ± 16.58 μmol/mol lysine; CEL: 516.61 ± 21.64 μmol/mol lysine; CML: 1131.87 ± 69.37 μmol/mol lysine and MDAL: 836.61 ± 45.11 μmol/mol lysine). *n* = 4 samples per group. *P < 0.05; **P < 0.01. AL, ad libitum; CR, Caloric restriction.

CR was associated with a reduction in steady-state levels of protein oxidative damage when measured by GC/MS, with glutamic semialdehyde (GSA), aminoadipic semialdehyde (AASA), carboxymethyl-lysine (CML), carboxyethyl-lysine (CEL) and malondialdehydelysine (MDAL) all significantly reduced in CR mice relative to AL mice (Fig. 3).

### CR induces changes in mitochondrial electron transport chain complexes and regulatory factors in liver

**Figure 4:**
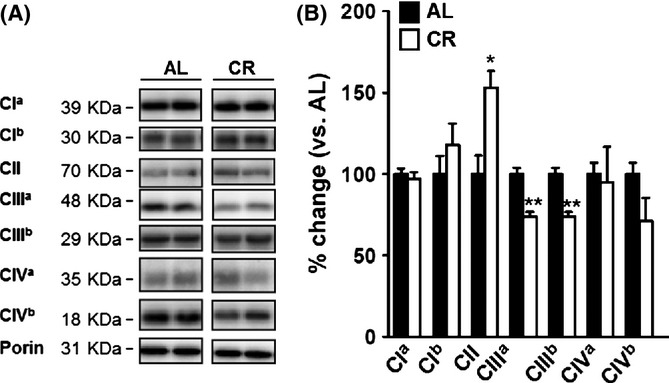
CR induces changes in the electron transport chain and regulatory factors. (A) Representative immunoblots showing the effect of CR in mitochondrial CI, II, III and IV (the levels of these components were estimated using representative subunits: CI^a^ = 39KDa (NDUFA9) subunit of complex I, CI^b^ = 30KDa (NDUFS3) subunit of complex I, CII = 70KDa (Flavoprotein) subunit of complex II, CIII^a^ = 48KDa (CORE2) subunit of complex III, CIII^b^ = 29KDa (Rieske iron-sulphur protein) subunit of complex III, CIV^a^ = COX1 subunit of complex IV and CIV^b^ = COX4 subunit of complex IV. (B) Relative amounts of CI-CIV in liver from AL and CR mice after adjusting for porin content and densitometric analyses. The mitochondrial complexes levels were related to porin. Values shown are means ± SEM from *n* = 4 samples per group. *P < 0.05 with respect to AL group by Student’s *t*-test. **P < 0.01. AL, ad libitum; CR, Caloric restriction.

Both increased protein proteolytic flux (either by proteasomal or autophagy-related mechanisms) and decreased lipid peroxidizability could help explain the lower levels of protein oxidative damage seen in liver from CR mice. However, these findings could also be explained by a decrease in oxidant production. Within most cells, the mitochondria are the major oxidant source, and so we evaluated whether mitochondrial electron transport chain components were changed following CR. We found that CR induced structural changes in liver mitochondria in mice (Fig. 4A,B), with higher levels complex II and lower levels of complex III relative to AL controls. No significant changes were observed in any other mitochondrial respiratory chain complexes (Fig. 4A,B).

### CR affects liver metabolome, specifically bioenergetic and tryptophan pathways

**Figure 5:**
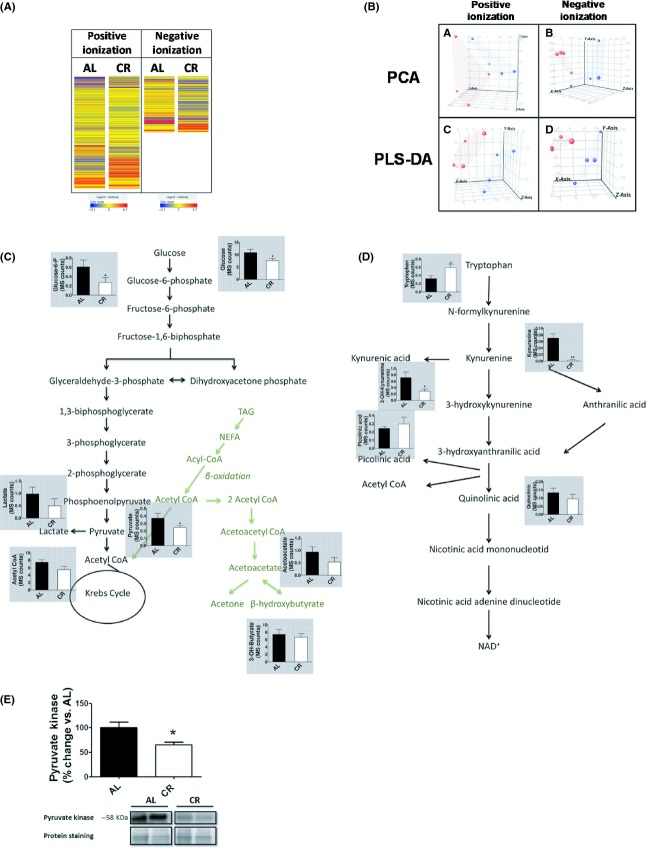
CR induces specific and marked changes in liver metabolome. (A) Heat map showing the molecular features (see main text for definition) found in liver from AL and CR mice in metabolomic analysis. Each line of this graphic represents an accurate mass ordered by retention time, coloured by its abundance intensity normalized to internal standard and baselining to median/mean across the samples. Individual scale maps for heat intensity are shown below each sample type. The scale from blue (lower abundance) to red (higher abundance) represents this normalized abundance in arbitrary units. *n* = 4 samples per group. (B) Principal component analysis (PCA) and Partial Least Square Discriminate Analysis (PLS-DA) graphs demonstrating the differentiation effect of diet in metabolomic profiles. Red spots represent samples from AL group and blue spots CR group. Explained variability of each representation in PCA is 66.05% (positive ionization) and 73.02% (negative ionization). Overall accuracy in PLS-DA analysis is 1 (for both positive and negative ionization). X: Principal component 1, Y: Principal component 2, Z: Principal component 3. CR induced changes in energy metabolism (C) and tryptophan (D) pathways. (E) CR decreased pyruvate kinase levels. Values shown are means ± SEM from *n* = 4 samples per group. *P < 0.05 with respect to AL group by Student’s *t*-test. AL, ad libitum; CR, Caloric restriction.

The CR linked mitochondrial changes could be associated with changes in global metabolism. For this reason, we analysed the effects of CR on the liver metabolome, by analysing the methanol-extractable metabolites present in liver homogenates by liquid chromatography linked to mass spectrometry. Using a nontargeted approach we detected 2983 metabolites (2166 in positive and 817 in negative ionization). This analysis revealed that CR significantly modifies the metabolome profile (Fig. 5A), with both PCA and PLS-DA approaches indicating that the diet could explain much of the variability observed between individual mice (Fig. 5B). The statistical analysis revealed that 210 metabolites differentiated between AL and CR animals, including several associated with glycolysis, fatty acid oxidation and tryptophan metabolism (Data S1). Focusing on energy metabolism pathways we detected lower levels of glucose, glucose-6-phosphate and pyruvate in CR liver samples (Fig. 5C). These changes in glycolytic pathway were partially confirmed using colorimetric methods (Figs S2 C–E). Moreover, and reinforcing the idea of lower glycolytic flux, the levels of pyruvate kinase enzyme were also decreased in CR mice (Fig. 5E). Concerning tryptophan oxidation pathway, tryptophan was increased and kynurenine and 3-hydroxykynurenine reduced by CR (Fig. 5D).

## Discussion

It is well established that CR increases lifespan and healthspan in a range of organisms (Anderson & Weindruch, 2010), although the mechanistic driver of these effects is currently unknown. Given the importance of lipids in cellular function and the relevant interaction between lipid composition, oxidative stress and aging (Hulbert *et al*., 2007; Naudí *et al*., 2013), we analysed the effect of CR on the hepatic lipidome of male C57BL/6 mice. Using a nontargeted approach and employing multivariate analysis (Jové *et al*., 2011), we demonstrated that CR induced marked changes in the liver lipidome profile. Our findings, using both unsupervised and supervised methods, revealed that diet (AL or CR) was the main factor explaining variability in lipid composition. The presented profiles included specific phospholipids, suggesting the selectivity of lipid-mediated mechanisms related to CR and aging. We also detected significant changes in TAG but not in total and free cholesterol and free fatty acid content following CR, suggesting that the free fatty acid released from triglycerides was immediately oxidized or exported to systemic circulation. The increased mitochondrial complex II content in CR animals further supports this idea of enhanced fatty acid oxidation rather than their export to the systemic circulation, in line with transcriptomic data showing enhanced fatty acid metabolism (Selman *et al*., 2006).

Based on the relationship between lipids and autophagy, (Ichimura *et al*., 2008) we analysed components of the autophagy pathway in CR mice. Macroautophagy, commonly known as autophagy, is an evolutionarily conserved process that helps to maintain proteostasis by digesting and removing damaged or unnecessary cellular components. The ubiquitin-like modifier LC3 covalently conjugates with PEs found in both inner and outer membrane of the phagophore, with this interaction being crucial for autophagosome maturation (Ichimura *et al*., 2008). Consequently, high levels of PEs are associated with high amount of phagophores. Furthermore, it has been recently described that pharmacological inhibition of autophagy increases TAG levels in hepatocytes, whereas autophagic inducers decrease their levels (Singh *et al*., 2009). The specific decreases in selected glycerolipids observed in CR mice also suggest higher uptake of intracellular lipid stores by autophagosomes. Cells store lipids in the form of lipid droplets, mainly formed by cholesterol and TAG. During autophagy, lipases break down TAG releasing free fatty acids which are consequently utilized by mitochondria to generate energy through beta-oxidation (Rodriguez-Navarro & Cuervo, 2010). This process requires cleavage of LC3 by Atg 4 to form cytosolic LC3-I. Subsequently, LC3-I undergoes conjugation to PE moiety by the Atg 7 and Atg 3 enzymes, to finally generate LC3-II, which is incorporated into the autophagosome membrane by the Atg 12-Atg 5-Atg 16 complex (Chiou *et al*., 2011). It has been shown that Atg 7 protein levels increased in skeletal muscle of 24-month-old rats under 8% CR but LC3-I and LC3-II levels were unaffected (Wohlgemuth *et al*., 2010). CR also increased levels of Atg7, but also increased the LC3-I/LC3-II ratio in rat heart (Wohlgemuth *et al*., 2007). However, CR decreased the levels of both LC3-I and II, and had no effect on Atg 7 levels in rat liver (Wohlgemuth *et al*., 2010). In agreement, we showed that both forms of LC3 were significantly decreased and Atg 7 was unaffected in mice under CR. This is compatible with the notion that decreased LC3 protein as a consequence of increased autophagic flux (more degradation) rather than lower conjugation of LC3-I to PE initiates autophagosome maturation. The elevation in PE levels in the liver of CR mice reinforced this hypothesis. The ratio between LC3-II and LC3-I is usually interpreted as a surrogate marker of autophagic flux: i.e. increased LC3-II/LC3-I increased autophagy flux (Shinmura *et al*., 2011). More autophagic flux could lead to more degradation of LC3-II and lower recycling to LC3-I. This mechanism could explain the present results, showing lower levels of both LC3-I and LC3-II in CR liver mice. p62 is a link between ubiquitinated proteins and autophagosome membranes through an interaction with LC3 (Gottlieb & Carreira, 2010), being commonly degraded by the autophagy–lysosome system. However, some studies suggest that LC3 is not essential for p62 recruitment to the autophagic structures (Itakura & Mizushima, 2011). It is also known that autophagy inhibition in hepatocytes and neurons leads to marked accumulation of p62 (Ichimura *et al*., 2008). 30% CR did not affect the levels of hepatic p62 although did decrease the levels of ubiquitinated proteins.

Single fatty acid profiling reveals that CR can change the relation between these fatty acids, lowering PUFA content and hereby decreasing the susceptibility to oxidation of the cellular membranes. In agreement, the present lipidomic study revealed lower levels of the lipoxidation product PGPC in CR mice. The fact that the CR did not influence the levels of other lipoxidation products such as HETE and 5-cholesten-3β-ol-7-one indicates the specificity of CR in modulating lipoxidative processes. These results are in accordance with an *in vivo* relevance of diminished DBI and PI index in CR. This can be attributed to redistribution in the type of unsaturation: CR increased MUFA levels in liver, whereas the levels of PUFA were decreased. No changes were observed in SFA levels. These results are in accordance with previous data in rat samples (Lambert *et al*., 2004; Gómez *et al*., 2007). While membrane fatty acid composition is strictly regulated, diet (as long as it has the minimum essential requirements –widely covered in our experimental conditions for both AL and CR groups) specifically appears to have limited influence on membrane composition (Abbott *et al*., 2012). However, this is not true for the balance between omega-3 polyunsaturates and omega-6 polyunsaturates because acyltransferase enzymes responsible for controlling membrane composition cannot differentiate between these two types of polyunsaturates (Abbott *et al*., 2012). In our study, diet polyunsaturated fat balance is maintained between our experimental groups. Consequently, the changes we report in lipid composition are likely due to a metabolic reprogramming following CR rather than alterations in lipid mass or composition (Chen *et al*., 2013).

Previous results (Gómez *et al*., 2007) demonstrated that 8.5% and 25% CR over 6–7 weeks decreased the levels of GSA, AASA, CEL, CML and MDAL (derived from protein direct oxidation, glycoxidation and lipoxidation processes) in rat liver. These five markers were significantly decreased after 8 weeks of CR in mice liver indicating the persistence of lower oxidative damage through species. These results could be attributed to either increased turn-over (both autophagic or proteasomal) and to decreased mitochondrial free radical production.

Many studies suggest that aging is caused by accumulation of reactive oxygen species-induced damage derived from mitochondria (Barja, 2013). Thus, to evaluate whether mitochondria were affected by CR, potentially leading to decreased oxidant production, the amounts of the four complexes of the respiratory chain were measured. Previous data showed that 7 weeks of 25% CR decrease complex I and IV and increase complex II and III in rat liver (Gómez *et al*., 2007). In this study, complex II levels were increased and complex I and IV were unaltered, while complex III was decreased. These differences in complex activity may be explained by a species-specific response to CR. Previous studies on the topology of free radical generation have shown that mitochondrial ROS production occurs at two of these four complexes, complex I and complex III (Barja, 2013). So, the decrease in complex III might contribute to a lower oxidant production. Furthermore, a key metabolic change during CR is a shift from carbohydrate metabolism to fat metabolism (Selman *et al*., 2006). The fatty acid-derived substrates enter the electron transport chain predominantly via complex II rather than complex I (Speakman & Mitchell, 2011) potentially explaining the higher levels of complex II found in CR liver samples reported in the present work.

Finally, we performed a metabolomic analysis to study the effect of 30% CR on liver metabolites. This approach demonstrated that CR induced marked changes in the metabolite profile of mice which reinforced our lipidomic data. Among a range of glycolytic metabolites detected, glucose, glucose-6-phosphate and pyruvate were decreased in CR mice as well as pyruvate kinase enzymes levels. It is known that CR reduces glycemia without changes in plasma pyruvate and lactate (Wijeyesekera *et al*., 2012). We also evaluated some final products of β-oxidation and our results indicated that CR did not alter the concentration of two ketone bodies (acetoacetate and hydroxybutyrate) in liver. CR in mice has previously been shown to elevate plasma acetoacetate and hydroxybutyrate levels (Selman *et al*., 2006; Wijeyesekera *et al*., 2012), suggesting export of these metabolites from the liver to plasma. Since very recent data show that ketone bodies export capacities are increased under fasting conditions via SLC16A (Hugo *et al*., 2012), our data may agree with increased β-oxidation under CR. The levels of acetyl-CoA, which can be derived from both glycolysis and β-oxidation pathways, was unaltered between AL and CR animals. Taking together, our results indicate that the glycolytic pathway is down-regulated, whereas the β-oxidation is up-regulated in liver under CR, consistent with the idea that the a metabolic switch occurs during CR (Selman *et al*., 2006).

Our untargeted approach revealed lower levels of kynurenine. It has been previously described that tryptophan pathway is affected by aging (Braidy *et al*., 2011). Consequently, we quantified and examined metabolites of this pathway. In CR mice, the levels of tryptophan were increased but 3-hydroxykynurenine and kynurenine were reduced relative to AL mice. No changes were observed in the levels of picolinic and quinolinic acids. These results suggest that CR is able to reverse the effects of aging in tryptophan pathway and open a new way to explore the mechanisms involved in healthy lifespan by CR. We should note that our experimental design (see ‘Experimental procedures’, ‘Animals and diet’ section) resulted in the CR animals potentially being fasted for slightly longer than the AL mice (1–2 h maximum). Consequently, we cannot discount at this time that this may have impacted on the differences in glycolytic and tryptophan metabolic pathways described under CR (see ‘Experimental procedures’, ‘Animals and diet’ section).

## Conclusion

Globally, the present data reveal that CR in mice induces a specific lipidomic and metabolomic signature in the liver along with an adaptation in mitochondrial function. Overall, these pathways could lead to decreased oxidative damage and contribute to the age-sparing measures of CR.

## Experimental procedures

### Animals and diet

Male C57BL/6 mice for the CR experiment were purchased at 4 weeks of age from a commercial breeder (Harlan Laboratories, Derby, UK). The 30% CR cohort underwent a step-down feeding regime as previously described, i.e. daily food intake was reduced to 90% of *ad libitum* (AL) fed mice at 14 weeks of age, 80% at 15 weeks and maintained at 70% of AL-fed mice intake from 16 weeks of age, i.e., 30% CR relative to AL controls (Selman *et al*., 2006). The CR food intake was adjusted according to the AL intake measured over the preceding week. Mice were maintained in groups of five and with the exception of the CR cohort, had AL access to chow (2018 Teklad Global 18% Protein Rodent Diet; Harlan Laboratories, UK). All animals were maintained under pathogen-free conditions within individually ventilated cages (Techniplast, Varese, Italy). CR mice were fed at ~09:00 hours each day following previously published protocols (Dhahbi *et al*., 2004; Selman *et al*., 2006; Wijeyesekera *et al*., 2012), with the hoppers of the CR mice always being empty by the following morning. AL and CR mice were killed by terminal anaesthesia (ip injection of fentanyl-fluanisone; Hypnorm, Janssen Animal Health, Beerse, Belgium) and benzodiazepine mix (Midazolam; Roche, Burgess Hill, UK) between 09:00 and 12:00 hours with AL and CR mice killed alternately each day to minimize potential circadian effects. CR mice were not fed in the morning immediately before death and AL mice were fasted overnight (17:00 hours) prior to the day of death (Selman *et al*., 2006; Wijeyesekera *et al*., 2012). After 2 months of dietary treatment, the animals were anesthetized and sacrificed. Tissues were removed and immediately stored at −80 °C until further analysis. All procedures were carried out following local ethical review (Imperial College London, London, UK), under a licence from the UK Home Office and followed the ‘principles of laboratory animal care’ (NIH Publication No. 86–23, revised 1985).

### Lipidome analysis

#### Sample processing

A quantity of 500 μg of liver were homogenized separately in a buffer containing 180 mm KCl, 5 mm 3-[N-morpholino]propanesulfonic acid, 2 mm ethylenediaminetetraacetic acid (EDTA), 1 mm diethylenetriaminepentaacetic acid and 1 mm butylated hydroxyl toluene, 10 mg mL^−1^ aprotinin, 1 mm phenylmethylsulfonyl fluoride, pH 7.3 with a Potter–Eljeveim device at 4 °C. Protein concentration was measured using the Lowry assay (Bio-Rad Laboratories, München, Germany) with bovine serum albumin as a standard. Total lipids from tissue samples were extracted with chloroform:methanol (2:1, v/v) in the presence of 0.01% butylated hydroxytoluene to avoid artifactual oxidation, as described previously (Gómez *et al*., 2007).

#### Fatty acid analysis by GC/MS

Fatty acyl groups in liver lipids were analysed as methyl esters derivatives by GC/MS as described previously (Gómez *et al*., 2007). Separation was performed in a SP2330 capillary column (30 m × 0.25 mm × 0.20 μm) in a Hewlett Packard 6890 Series II gas chromatograph. A Hewlett Packard 5973A mass spectrometer was used as detector in the electron-impact mode. Identification of fatty acyl methyl esters was made by comparison with authentic standards and on the basis of mass spectra. Results are expressed as mol%. The following fatty acyl indices were also calculated: SFA; UFA; MUFA; PUFA from n-3 and n-6 series (PUFAn-3 and PUFAn- 6); average chain length (ACL) = [(Σ%Total_14_ × 14) + (Σ%Total_16_ × 16) + (Σ%Total_18_ × 18) + (Σ%Total_20_ × 20) + (Σ%Total_22_ × 22)]/100; double bond index (DBI) = [(1 × Σ mol% monoenoic) + (2 × Σ mol% dienoic) + (3 × Σ mol% trienoic) + (4 × Σ mol% tetraenoic) + (5 × Σ mol% pentaenoic) + (6 × Σ mol% hexaenoic)], and peroxidizability index (PI) = [(0.025 × Σ mol% monoenoic) + (1 × Σ mol% dienoic) + (2 × Σ mol% trienoic) + (4 × Σ mol% tetraenoic) + (6 × Σ mol% pentaenoic) + (8 × Σ mol% hexaenoic)].

#### QTOF-based lipidome analysis

For LC-Q-TOF-based lipid molecular species analyses, 20 μL of liver homogenated (containing 200 μg of protein) was diluted to 250 μL with a phosphate buffer. After adding representative internal standards lipid extraction with chloroform:methanol (2:1; v/v) was done. The chloroform phase was extracted and evaporated using a Speed Vac (Thermo Fisher Scientific, Barcelona, Spain) and resuspended in chloroform:methanol (1:3, v/v).

Lipid extracts were subjected to mass spectrometry using a HPLC 1290 series coupled to an ESI-Q-TOF MS/MS 6520 (Agilent Technologies, Barcelona, Spain) as described previously (Jové *et al*., 2013).

Targeted lipidomic analysis was performed using the same chromatographic and spectrometric method as untargeted approach. MassHunter Qualitative Analysis Software (Agilent Technologies) was employed for integration and extraction of peak intensities of the different lipid species. We searched for (i) different free fatty acids as docosahexaenoic acid, oleic acid, linolenic acid, stearic acid, arachidonic acid, lauric acid, palmitic acid, capric acid and myristic acid, (ii) cholesterol and (iii) different soluble oxidative stress-related markers as 10-hydroxy-docosahexaenoic, 17-hydroxy-docosahexaenoic, 8-isoprostaglandin F2α, 13- HODE, 9-HODE, HODE-cholesteryl ester, HETE, PGPC, 1PazPC, 4-hydroxynonenal, 10-nitrooleate, cholesterol-5α,6α-epoxide, 5-cholesten-3β-ol-7-one, 7β-hydroxycholesterol, resolvin D1, cholesteryl linoleate hydroperoxide and cholesteryl linoleate.

The m/z values used for the quantification of lipid molecules detected were as follows: m/z 245.2486 [M−H]^−^ for oleic acid, m/z 283.2643 [M−H]^−^ for stearic acid, m/z 255.233 [M−H]^−^ for palmitic acid, m/z 279.233 [M−H]^−^ for linoleic acid, m/z 303.233 [M−H]^−^ for arachidonic acid, m/z 327.233 [M−H]^−^ for docosahexaenoic acid, m/z369.3487 [M+H]^+^+[−H_2_O] for cholesterol; m/z 401.3414 [M+H]^+^ for 5-cholesten-3β-ol-7-one, m/z 321.2424 [M+H]^+^ for HETE and m/z 610.3715 [M+H]^+^ for PGPC.

### Protein electrophoresis and western blot analysis

Samples were homogenized in a buffer containing 180 mm KCl, 5 mm 3-[N morpholino]propanesulfonic acid, 2 mm ethylenediaminetetraacetic acid (EDTA), 1 mm diethylenetriaminepentaacetic acid and 1 mm butylated hydroxyl toluene, 10 mg mL^−1^ aprotinin, 1 mm phenylmethylsulfonyl fluoride, pH 7.3 with a Potter– Eljeveim device at 4 °C. Immunodetection was performed using specific antibodies (Table S2). An antibody to porin as a control for total mitochondrial mass control was used. Alternatively, ponceau staining or beta-actin, as a control for total protein charge was also used to determine the proportion of protein levels referred to total protein content. Appropriate peroxidase-coupled secondary antibodies and chemiluminescence HRP substrate (Millipore, Billerica, MA, USA) were used for primary antibody detection. Signal quantification and recording were performed with ChemiDoc equipment (Bio-Rad Laboratories, Inc., Barcelona, Spain). Control experiments showed that omission of primary or secondary antibody addition produced blots with no detectable signal.

### Oxidation-derived protein damage markers measurements in liver proteins by GC/MS

Five structurally identified markers of oxidative damage, representative of three major oxidative modifications of proteins (the direct oxidation-derived-specific protein carbonyls, GSA and AASA, the glycoxidation-derived CEL and the lipoxidation-derived CML and MDAL) were measured by gas chromatography/mass spectrometry (GC/MS) as described previously (Pamplona *et al*., 2005). GC/MS analyses were carried out on a Hewlett-Packard model 6890 gas chromatograph equipped with a 30 m HP-5MS capillary column (30 m × 0.25 mm × 0.25 μm) coupled to a Hewlett-Packard model 5973A mass selective detector (Hewlett-Packard Española, S.A., Barcelona, Spain). Analytes were detected by selected ion-monitoring GC/MS. The ions used were as follows: lysine and [^2^H_8_]lysine, m/z 180 and 187 respectively; 5-hydroxy-2-aminovaleric acid and [^2^H_5_]5-hydroxy-2-aminovaleric acid (stable derivatives of GSA), m/z 280 and 285 respectively; 6-hydroxy-2-aminocaproic acid and [^2^H_4_]6-hydroxy-2-aminocaproic acid (stable derivatives of AASA), m/z 294 and 298 respectively; CML and [^2^H_4_]CML, m/z 392 and 396 respectively; CEL and [^2^H_4_]CEL, m/z 379 and 383 respectively; and MDAL and [^2^H_8_]MDAL, m/z 474 and 482 respectively. The amounts of products were expressed as the ratio μmol glutamic semialdehyde, AASA, CML, CEL or MDAL/mol lysine.

### Metabolome analysis

Metabolites were extracted from liver homogenates samples with methanol according to previously described methods (Wikoff *et al*., 2008). The supernatant were recovered, evaporated using a Speed Vac (Thermo Fisher Scientific) and resuspended in water 0.4% acetic acid and 2 ng mL^−1^ of docosahexaenoic acid in methanol as a internal standard (1:1, v/v).

For the metabolomic studies, an Agilent 1290 LC system coupled to an ESI-Q-TOF MS/MS 6520 instrument (Agilent Technologies) was used. In all cases, 2 μL of extracted sample was applied onto a reversed-phase column (Zorbax SB-Aq 1.8 μm 2.1 × 50 mm; Agilent Technologies) equipped with a precolumn (Zorba-SB-C8 Rapid Resolution Cartridge 2.1 × 30 mm 3.5 μm; Agilent Technologies) with a column temperature of 60 °C. The flow rate was 0.6 mL min^−1^. Solvent A was composed of water containing 0.2% acetic acid and solvent B was composed of methanol 0.2% acetic acid. The gradient started in 2% B and increased to 98% B in 13 min and hold at 98% B for 6 min. Pot-time was established in 5 min.

Data were collected in positive and negative electrospray mode TOF operated in full-scan mode at 50–1600 m/z in an extended dynamic range (2 GHz), using N2 as the nebulizer gas (10 L min^−1^, 350 °C) as described previously (Gonzalo *et al*., 2012). All molecules were adjusted to internal standard ionizable in both positive and negative modes (deuterated docosahexaenoic acid).

Targeted metabolomic analysis was performed using the same chromatographic and spectrometric method as untargeted approach. MassHunter Qualitative Analysis Software (Agilent Technologies) was employed for integration and extraction of peak intensities of the different metabolic species. The m/z values used for quantification were as follows: m/z 219.0226 [M+K]^+^ for glucose, 298.9865 [M+K]^+^ for glucose-6-phosphate, 111.0053 [M+Na]^+^ for pyruvate, m/z 89.0264 [M−H]^−^ for lactate, m/z 810.133 [M−H]^−^ for Acetyl-coA, m/z 85.0260 [M+H]^+^+[−H_2_O] for acetoacetate, m/z 85.0299 [M−H]^−^+[-H_2_O] for 3-hydroxybutyrate, m/z 205.1023 [M+H]^+^ for tryptophan, m/z 225.0882 [M+H]^+^ for 3-hydroxykynurenine, m/z 189.0644 [M−H]^−^+[−H_2_O] for kynurenine, m/z 124.0393 [M+H] for picolinic acid and m/z 167.0477 [M+NH_4_]^+^+[−H_2_O] for quinolinic acid. All molecules were adjusted to internal standard ionizable in both positive and negative modes (deuterated docosahexaenoic acid).

### Biochemical analyses

Glucose, lactate, triglycerides, cholesterol content and pyruvic acid were quantified by enzymatic colorimetric reactions using commercial kits (Spinreact, Girona, Spain; Abcam, Cambridge, UK).

### Statistics

All statistics were performed using the spss software (spss Inc., Chicago, IL, USA). All values were expressed as means ± standard error of the mean (SEM). Comparisons between AL and CR animals were statistically analysed with Student’s *t-*tests. The minimum level of statistical significance was set at *P* < 0.05 in all the analyses.

## Supporting Information

**Fig. S1.** CR does not affect the levels of free fatty acids and cholesterol in the liver of male C57BL/6 mice. **Fig. S2.** Liver metabolites measured by colorimetric methods. **Table S1.** Fatty acyl composition (mol%) of total lipids in liver from male C57/BL6 mice. **Table S2.** Antibodies used for Western Blot experiments.

**Data S1.** Lipid species in the liver differ between AL and CR animals.
