# Supplementary material for: Caloric restriction reveals a metabolomic and lipidomic signature in liver of male mice
Source: Aging Cell. 2014 Jul 23;13(5):828–37. doi: 10.1111/acel.12241 (PMC4331741; doi:10.1111/acel.12241)
Supplement: Supplementary file 1 — Fig. S1. CR does not affect the levels of free fatty acids and cholesterol in the liver of male C57BL/6 mice. Fig. S2. Liver metabolites measured by colorimetric methods. Table S1. Fatty acyl composition (mol%) of total lipids in liver from male C57/BL6 mice. Table S2. Antibodies used for Western Blot experiments. [file acel0013-0828-sd1.docx]

**Supporting Information**

**Supplementary Figure 1.** CR does not affect the levels of free fatty acids and cholesterol in liver mice. Values shown are means ± SEM from *n*=4 samples per group.

**Supplementary Figure 2.** . Liver metabolites measured by colorimetric methods. Values shown are means ± SEM from *n*=4 samples per group.

**
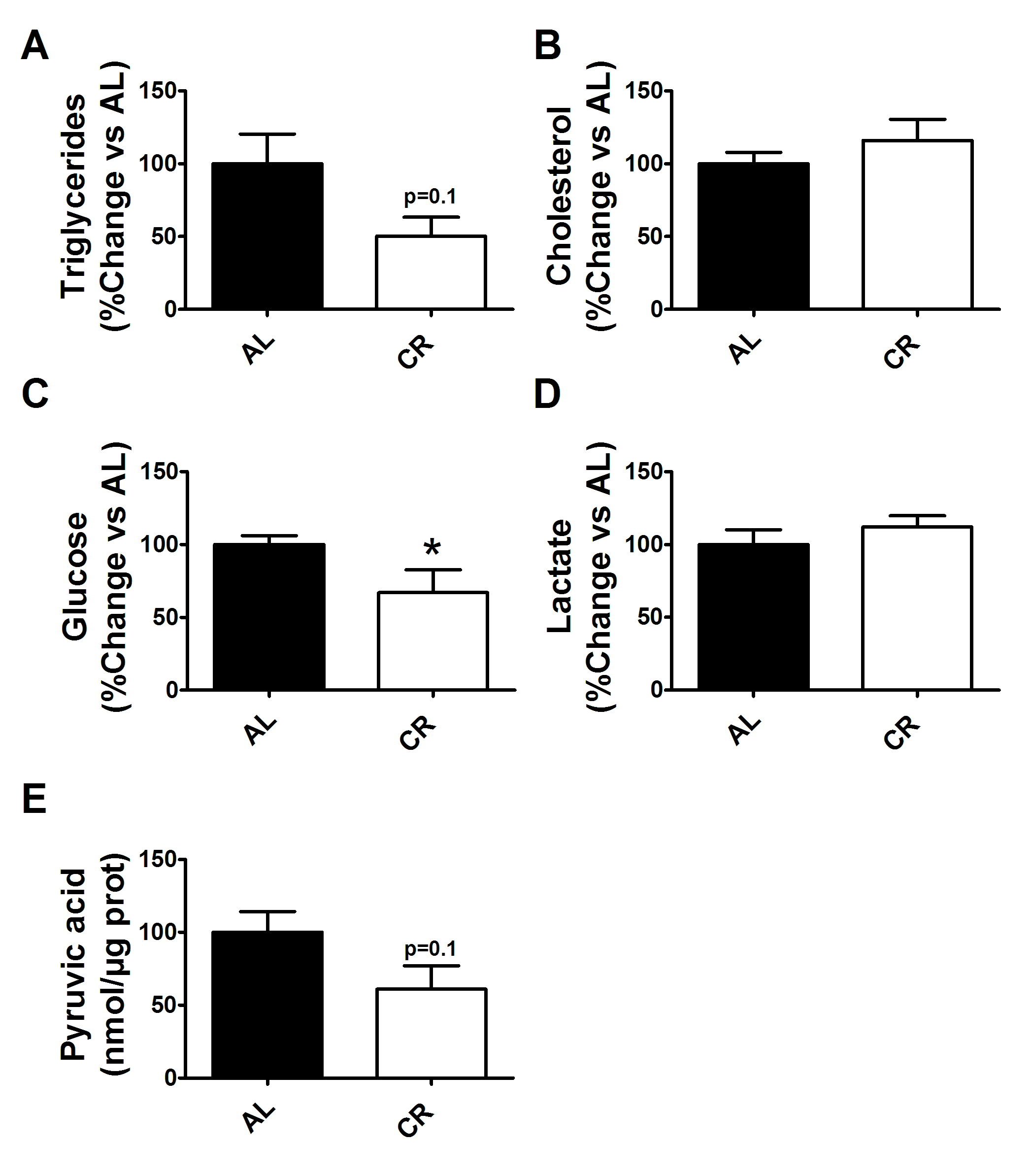
**

# Supplementary Table 1. Fatty acyl composition (mol %) of total lipids in liver from male C57/BL6 mice.

|  | **AL** | **CR** | **P** |
| --- | --- | --- | --- |
|  |  |  |  |
| **14:0** | 0.27±0.05 | 0.24±0.003 | 0.573 |
| **16:0** | 26.68±0.26 | 27.05±0.73 | 0.660 |
| **16:1n-7** | 1.88±0.21 | 3.99±0.37 | 0.003 |
| **18:0** | 9.90±1.13 | 7.53±0.04 | 0.081 |
| **18:1n-9** | 19.09±1.91 | 23.80±0.72 | 0.050 |
| **18:2n-6** | 19.08±0.81 | 16.29±0.16 | 0.016 |
| **18:3n-3** | 0.54±0.17 | 0.40±0.07 | 0.487 |
| **20:0** | 1.94±0.36 | 1.64±0.06 | 0.459 |
| **20:3n-6** | 0.86±0.08 | 1.17±0.02 | 0.010 |
| **20:4n-6** | 5.83±0.59 | 5.05±0.008 | 0.233 |
| **20:5n-3** | 1.16±0.07 | 1.64±0.14 | 0.028 |
| **22:0** | 0.91±0.18 | 1.08±0.17 | 0.519 |
| **22:5n-3** | 0.83±0.13 | 0.93±0.02 | 0.464 |
| **22:6n-3** | 10.97±0.39 | 9.13±0.52 | 0.032 |
|  |  |  |  |
| **ACL** | 18.12±0.03 | 18.00±0.009 | 0.023 |
| **SFA** | 39.73±1.99 | 37.56±0.58 | 0.337 |
| **UFA** | 60.26±1.99 | 62.43±0.58 | 0.337 |
| **MUFA** | 20.98±2.12 | 27.80±1.10 | 0.029 |
| **PUFA** | 39.28±0.20 | 34.63±0.52 | 0.001 |
| **PUFAn-6** | 25.77±0.15 | 22.51±0.19 | 0.001 |
| **PUFAn-3** | 13.51±0.34 | 12.11±0.71 | 0.131 |
|  |  |  |  |
| **DBI** | 162.50±0.87 | 153.03±2.43 | 0.011 |
| **PI** | 145.50±4.40 | 128.90±4.79 | 0.043 |
|  |  |  |  |

# Values are means ± SEM from n=4 samples per group.

**Supplementary Table 2.** Antibodies used for Western Blot experiements

| **Antibody** | **Reference** | **Dilution** |
| --- | --- | --- |
| 39KDa subunit of complex I (NDUFA9) | A21344, Molecular Probes, Madrid, Spain | 1:1000 |
| 30KDa subunit of complex I (NDUFS3) | A2134, Molecular Probes, Madrid, Spain 3 | 1:1000 |
| 70KDa subunit (Flavoprotein) of complex II | A11142, Molecular Probes, Madrid, Spain | 1:1000 |
| 48KDa (CORE 2) subunits of complex III | A11143, Molecular Probes, Madrid, Spain | 1:1000 |
| 29KDa (Rieske iron-sulfur protein) subunits of complex III | A21346, Molecular Probes, Madrid, Spain | 1:1000 |
| COXI subunit of complex IV | A6403, Molecular Probes, Madrid, Spain | 1:1000 |
| COXIV subunit of complex IV | MS407; Mitosciences, Eugene, Oregon, USA | 1:1000 |
| AIF | a7549, Sigma Aldrich, S. Louis, MO, USA | 1:1000 |
| PGC-1α | 101707, Cayman Chemical, Ann Arbor, Michigan, USA | 1:1000 |
| UCP2 | ab32592, Abcam, Cambridge, UK | 1:125 |
| SIRT1 | sc-19857, Santa Cruz Biotechnology, Inc., CA, USA | 1:200 |
| Atg7 | 3615, ProScience, Woburn, MA, USA | 1:1000 |
| LC-3b | 2775, Cell Signaling, Danvers, MA, USA | 1:2000 |
| p62 | PM045, MBL International Corp., Woburn, MA, USA | 1:1000 |
| ubiquitin | U5379, Sigma Aldrich, S. Louis, MO, USA | 1:100 |
| AMPK | I2532, Cell Signaling, Danvers, MA, USA | 1:1000 |
| AMPKp | 2535, Cell Signaling, Danvers, MA, USA | 1:1000 |
| PKM2 | Ab38237, Abcam, Cambridge, UK | 1:500 |
| porin | A31855, Molecular Probes, Madrid, Spain | 1:5000 |
| beta-actin | ab20272, Abcam, Cambridge, UK | 1:5000 |
